# Apple chlorotic fruit spot viroid: a putative new pathogenic viroid on apple characterized by next-generation sequencing

**DOI:** 10.1007/s00705-019-04420-9

**Published:** 2019-10-09

**Authors:** Thomas Leichtfried, Stefanie Dobrovolny, Helga Reisenzein, Siegrid Steinkellner, Richard A. Gottsberger

**Affiliations:** 1 Institute for Sustainable Plant Protection, Austrian Agency for Health and Food Safety, 1220 Vienna, Austria; 2 Institute of Plant Protection, University of Natural Resources and Life Sciences Vienna, 3430 Tulln an der Donau, Austria; 3 Institute for Food Safety, Austrian Agency for Health and Food Safety, 1220 Vienna, Austria

## Abstract

Viroid-like symptoms were observed in 2016 on apple fruits of the cultivar “Ilzer Rose” in southern Burgenland, Austria. Preliminary molecular biological investigations indicated that the symptoms were caused by a new unknown viroid. Therefore, new primers were designed, and the whole genome sequence of the viroid (354 nt) was determined by next-generation amplicon sequencing using the Illumina MiSeq^®^ platform (San Diego, California, USA). The viroid secondary structure has a rod-like conformation and contains conserved regions (the TCR, CCR upper strand, and CCR lower strand) that are characteristic of members of the genus *Apscaviroid*. Based on our results and the demarcation criteria for viroids, the tentatively named “apple chlorotic fruit spot viroid*”* should be considered a putative new member of the genus *Apscaviroid*.

**Electronic supplementary material:**

The online version of this article (10.1007/s00705-019-04420-9) contains supplementary material, which is available to authorized users.

## Introduction

**Fig. 1:**
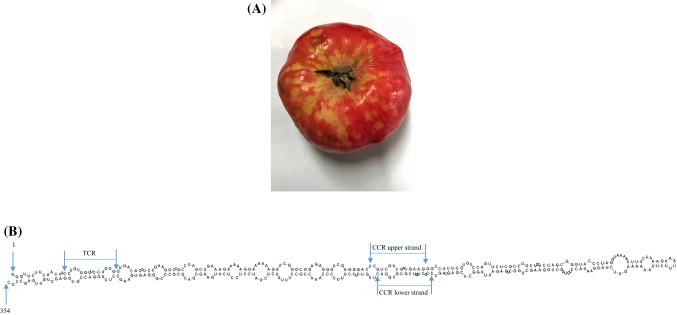
(A) Yellow and chlorotic fruit spots and peak-like symptoms on the skin of apples of cv. “Ilzer Rose” caused by ACFSVd. (B) Nucleotide sequence and proposed secondary structure of ACFSVd. Structures are represented for the lowest-energy form at 37 °C predicted using the model of Turner and Mathews [18], created using Geneious® software (Version 10.1.3). The first and the last nucleotide of ACFSVd, the TCR and the CCR lower and upper strands are indicated by arrows

Apple is an important fruit with a high economic impact and is also the most important fruit crop in Austria [1]. Viroids can cause evident fruit symptoms on pome fruits and can cause economic losses by reducing fruit yield and quality, thus resulting in unmarketable fruits [2]. More than 30 plant-pathogenic viroids are known thus far. In apple, three plant-pathogenic viroids, all belonging to the genus *Apscaviroid*, have been described: apple scar skin viroid (ASSVd), apple dimple fruit viroid (ADFVd), and the unassigned apple fruit crinkle viroid (AFCVd) [3]. Recently, a new technique, next-generation sequencing (NGS), was developed, and this can be used as an important tool for the discovery [4–7], detection, identification, and characterization [8] of new plant viroids and viruses [9, 10]. The major advantage of NGS is the ability to generate an immense amount of data in a short time; thus, it is a cost-effective method to obtain extensive genome information rapidly. In 2016, viroid-like symptoms were observed on apple fruits of the local cultivar “Ilzer Rose” in the Austrian province Burgenland (Fig. 1A). In this paper, we report the detection and identification of the whole genome of this putative new pathogenic viroid on apple using NGS amplicon sequencing.

## Materials and methods

For the first screening, total RNA was extracted directly from symptomatic apple skins (cv. “Ilzer Rose”) using an RNeasy Plant Mini Kit (QIAGEN, Hilden, Germany), and the generic primers PBCV100C and PBCV194H were used for the detection of pome fruit viroids [11]. Based on the generic sequences, two primer sets were designed to sequence the whole genome and to confirm the circular form of the unknown viroid by NGS (primer set 1, ACFSVd_F1 [5′-CTGAGATTGGCTCGAGGAGTCC-3′] and ACFSVd_R1 [5′-GCGAGTTCTGGACACGAGAG-3′]; primer set 2, ACFSVd_F2 [5′-CCGCCTTTTTCTCTATCCTC-3′] and ACFSVd_R2 [5′-AGCAGGCGAGAACTGGACAC-3′]) (Supplementary Fig. S1).

DNA library preparation was performed in a two-step PCR process with a dual-indexing principle according the “16S Metagenomics Sequencing Library Preparation” protocol from Illumina and modifications described by Dobrovolny et al. [12] The sequencing reaction was performed on an Illumina MiSeq^®^ platform (San Diego, California, USA) using a 300-cycle MiSeq® Reagent Kit. Further data analysis and processing steps were implemented in the Galaxy workflow [12, 13]. The *de novo* assembly of the obtained sequences was performed using Geneious^®^ software (Version 10.1.3).

Phylogenetic analysis was carried out based on whole genome sequences (range, 269-401 nt) of members of the genus *Apscaviroid*. The reference sequences, except ACFSVd, were retrieved from NCBI GenBank. A multiple alignment was generated using the Geneious algorithm (similar to the Clustal W algorithm) in Geneious^®^ software (Version 10.1.3).

## Results and discussion

**Fig. 2:**
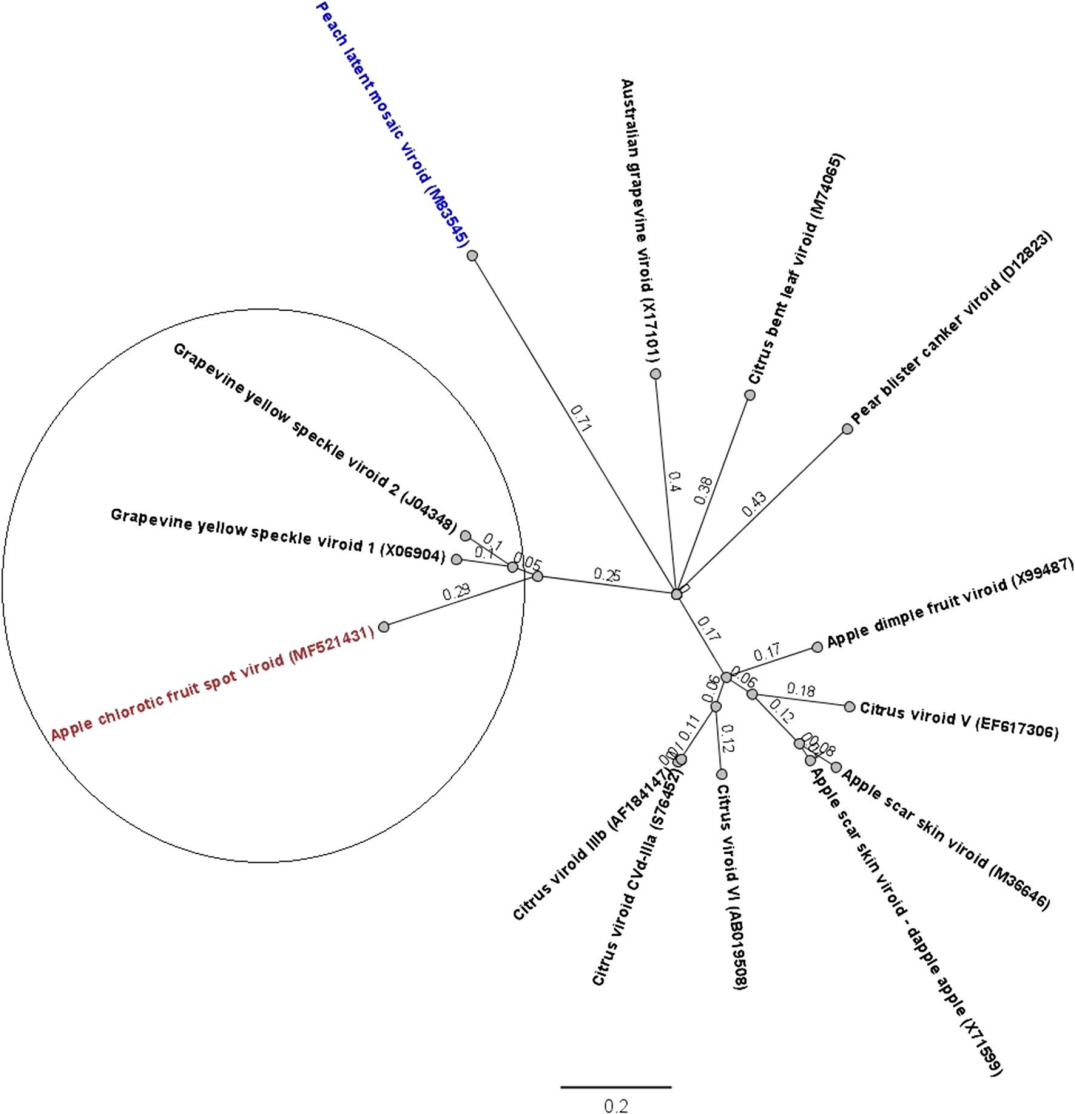
Phylogenetic analysis of ACFSVd including all assigned members of the genus *Apscaviroid* and peach latent mosaic viroid as an outgroup (labelled in blue). The multiple sequence alignment was generated using Geneious® software (version 10.1.3). The phylogenetic tree was constructed by the neighbour-joining (NJ) clustering method [19], and 5,000 bootstrap replicates were performed. The genetic distance was calculated using the Tamura-Nei model [20]. ACFSVd is indicated in red and bold. The scale represents 0.2 nucleotide substitutions per site. The circle indicates the cluster of adjacent relatives. GenBank accession numbers are shown in brackets

After the reads were processed via a quality check and the Galaxy workflow, the number of sequences obtained from the Illumina MiSeq^®^ platform with primer set 1 was 145,679 reads, while the number of sequences obtained with primer set 2 was 21,160 reads. The operational taxonomic unit (OTU) with the largest number of identical sequences was used to assemble the genome sequence of ACFSVd using Geneious^®^ software (Version 10.1.3). The whole-genome data have been deposited in the databases provided by NCBI (accession no. MF521431.2). The whole genome of ACFSVd is 354 nt long (Fig. 1B). The circular RNA molecule consists of 76 A (21.5 %), 95 C (26.8 %), 97 G (24.3 %) and 86 U (24.3 %) with a G+C content of 54.2 % and a free energy of -147.40 kcal/mol (Fig. 1B). The characteristic features of the secondary structure of the genome of members of the genus *Apscaviroid* are the terminal conserved regions (TCRs), the upper and lower central conserved regions (CCRs), and a quasi-rod-like conformation (Fig. 1B) [14]. No mismatches were observed between the TCR region and CCR upper strand of ACFSVd and those of the apscaviroid ASSVd type reference (NC_001340). There was only one mismatch on the upper strand (Table 1). The sequence was compared to sequences in the NCBI GenBank database by BLASTn analysis. This RNA molecule is most similar to grapevine speckle viroid 2 (80.8 %) (accession no. FJ597935.1) and grapevine speckle viroid 1 (76.7 %) (accession no. KF007313.1), but the similarity is limited to a short fragment. Phylogenetic analysis supported the BLAST results. ACFSVd formed a monophyletic group with grapevine speckle viroid 1 and grapevine speckle viroid 2, and the genetic distance revealed that ACFSVd is clearly a new member of the genus *Apscaviroi*d (Fig. 2). ACFSVd differs from other confirmed apple viroids in the genus *Apscaviroid*, such as ASSVd and ADFVd, in the symptoms it causes in apple fruits. The symptoms of ACFSVd infection are yellow chlorotic spots and pronounced peak-like deformations on apple fruits (Fig. 1A). In contrast, ASSVd causes scarred skin or speckle symptoms on apple fruits [15], and ADFVd induces yellow-green spots, which are sometimes slightly depressed (dimpled) and scattered mainly around the calyx [16]. To test the transmissibility of the RNA molecule, scions were grafted from the symptomatic apple tree cultivar Ilzer Rose to three apple trees of the cultivar Gala. These trees were tested positive distant from the graft after half a year using RT-PCR with the ACFSVd-specific primers ACFSVd-F (5′-CTAGTCGCGCGGACTTGTCTC-3′) and ACFSVd-R (5′-CGAGAACTGGACACGAGAGG-3′).

**Table 1:** Terminal conserved region (TCR) and central conserved region (CCR) sequences of ACFSVd and ASSVd, the type member of the genus *Apscaviroid*. One mismatch (underlined and bold) was observed in the CCR lower strand. GenBank accession numbers are shown in brackets

	TCR	CCR upper strand	CCR lower strand
ASSVd (NC_001340)	CNNGNGGUUCCUGUGG	UCGUCGUCGACGAAGG	CCGCUAGUCGAGCGGAC
ACFSVd (MF521431.2)	CCUGUGGUUCCUGUGG	UCGUCGUCGACGAAGG	CCGCUAGUCG**C**GCGGAC

Analyses of the complete nucleotide sequence indicated that ACFSVd is a putative new viroid in the genus *Apscaviroid* according to the demarcation criteria from the International Committee on Taxonomy of Viruses (ICTV) [14, 17]. The whole genome sequence was used to generate a phylogenetic tree. The evaluation of the data showed that the “closest” relatives are grapevine speckle viroid 1 and 2. ACFSVd is transmissible by grafting. Further pathways are still under investigation.

In summary, in this study, a putative new unknown pathogenic viroid of apple was discovered and identified using NGS technology. The species demarcation criteria for viroids indicate that ACFSVd should be regarded as a new distinct member of the genus *Apscaviroid*.

## Electronic supplementary material

Below is the link to the electronic supplementary material.

Supplementary material 1 (DOC 165 kb)
