# Supplementary material for: Apple chlorotic fruit spot viroid: a putative new pathogenic viroid on apple characterized by next-generation sequencing
Source: Arch Virol. 2019 Oct 9;164(12):3137–40. doi: 10.1007/s00705-019-04420-9 (PMC6823291; doi:10.1007/s00705-019-04420-9)
Supplement: Supplementary file 1 — Supplementary material 1 (DOC 165 kb) [file 705_2019_4420_MOESM1_ESM.doc]

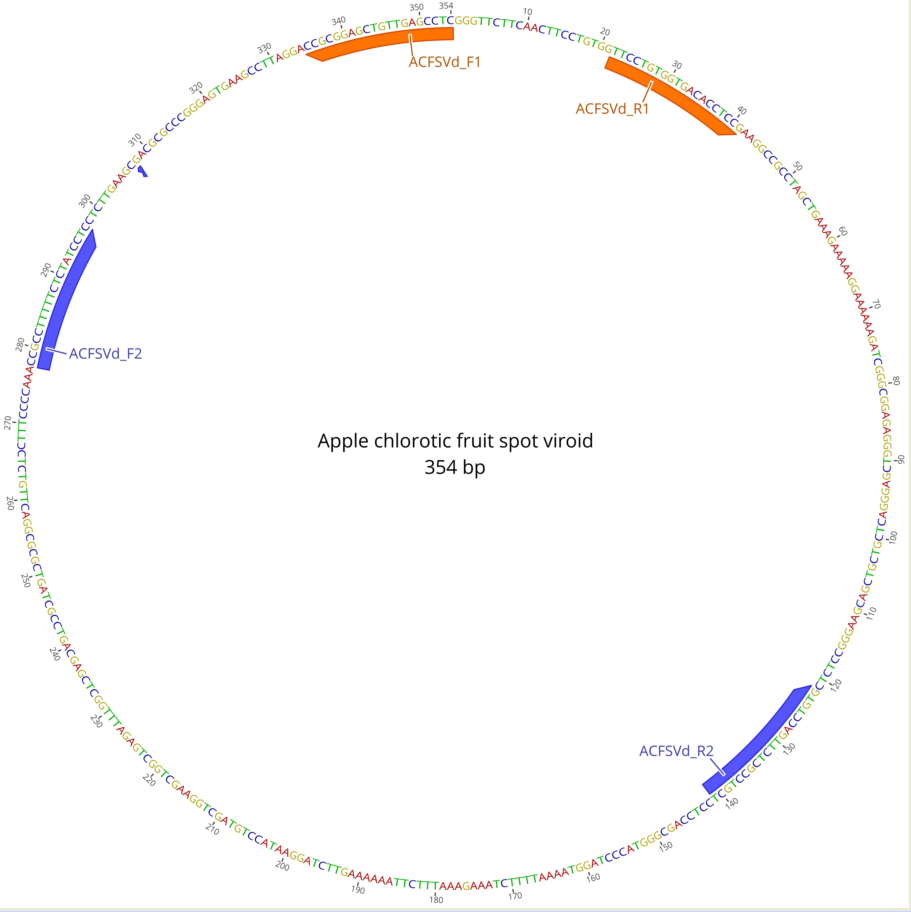


**Supplementary Figure S1.** Circular RNA molecule of ACFSVd. The used primer pairs for sequencing the whole genome and to confirm the circular form are labelled in orange (Primerset 1) and blue (Primerset 2).
